# Mixed infection and clonal representativeness of a single sputum sample in tuberculosis patients from a penitentiary hospital in Georgia

**DOI:** 10.1186/1465-9921-7-99

**Published:** 2006-07-17

**Authors:** Isdore C Shamputa, Levan Jugheli, Nikoloz Sadradze, Eve Willery, Françoise Portaels, Philip Supply, Leen Rigouts

**Affiliations:** 1 Prince Leopold Institute of Tropical Medicine, Mycobacteriology Unit, Nationalestraat 155, B-2000 Antwerp, Belgium; 2 Tropical Diseases Research Centre, Microbiology Unit, P. O. Box 71769, Ndola, Zambia; 3 International Committee of the Red Cross, 4, Kedia Str. 380054, Tbilisi, Georgia; 4 Laboratoire des Mécanismes Moléculaires de la Pathogenèse Bactérienne, INSERM U629, Institut de Biologie/Institut Pasteur de Lille, Lille, France

## Abstract

**Background:**

Studies on recurrent tuberculosis (TB), TB molecular epidemiology and drug susceptibility testing rely on the analysis of one *Mycobacterium tuberculosis *isolate from a single sputum sample collected at different disease episodes. This scheme rests on the postulate that a culture of one sputum sample is homogeneous and representative of the total bacillary population in a patient.

**Methods:**

We systematically analysed several pre-treatment isolates from each of 199 smear-positive male adult inmates admitted to a prison TB hospital by standard IS*6110 *DNA fingerprinting, followed by PCR typing based on multiple loci containing variable number of tandem repeats (VNTRs) on a subset of isolates. Drug susceptibility testing (DST) was performed on all isolates for isoniazid, rifampicin, streptomycin and ethambutol.

**Results:**

We found mixed infection in 26 (13.1%) cases. In contrast, analysis of a single pre-treatment isolate per patient would have led to missed mixed infections in all or 14 of these 26 cases by using only standard DNA fingerprinting or the PCR multilocus-based method, respectively. Differences in DST among isolates from the same patient were observed in 10 cases, of which 6 were from patients with mixed infection.

**Conclusion:**

These results suggest that the actual heterogeneity of the bacillary population in patients, especially in high TB incidence settings, may be frequently underestimated using current analytical schemes. These findings have therefore important implications for correct interpretation and evaluation of molecular epidemiology data and in treatment evaluations.

## Background

Tuberculosis (TB) has been traditionally assumed to result from a single infection with a single *Mycobacterium tuberculosis *strain, and this infection is thought to confer immunity to additional infections. Therefore, a recurrence of disease has been most often considered to be caused by endogenous reactivation of the strain that caused the original infection (relapse). Consequently, almost all current analytical schemes of clinical or research relevance are still based on examination of single isolates of given disease episodes, with implicit assumption that this isolate is representative of an homogeneous bacillary population.

This model of homogeneous infection has been revised by several studies using strain typing methods, which have demonstrated the occurrence of infection with clonally distinct strains, especially in high-incidence settings [1-3]. Both human immunodeficiency virus (HIV)-negative and HIV-positive individuals can be infected with more than one strain during a given disease episode (mixed infection), or re-infected by a second *M. tuberculosis *strain during a recurrent episode (exogenous re-infection). Such findings have important implications for control programs, vaccine development, evaluation of treatment regimens [4], and for epidemiological interpretation [3,5].

However, these studies of re-infection and mixed infection have so far been conducted by analysing the genotypes of the isolate from one sputum specimen from the initial and recurrent episodes or from one given episode, respectively [6-8]. Such approaches discount the old postulate that bacilli sequestered at different pulmonary infection sites are not necessarily released in the sample provided. Therefore, analysis of a single isolate might underestimate the actual heterogeneity of the bacillary population in the host. Conversely, the consequences of clonal heterogeneity on the representativeness of a single isolate have remained unknown hitherto.

Here, we have prospectively evaluated both the frequency of mixed infections and the clonal heterogeneity among clinical isolates from the same patient by analysing at least two pre-treatment isolates from each of 199 TB patients from a prison TB hospital in Georgia, consecutively enrolled over a period of three years. These isolates were analysed by using standard IS6*110*-restriction fragment length polymorphism (RFLP) genotyping as a first-line screening, followed by typing based on PCR amplification of 15 different loci containing mycobacterial interspersed repetitive unit-variable number of tandem repeats (MIRU-VNTRs) for independent confirmation of simultaneous presence of multiple strains. The implications of the results for current analytical schemes of drug susceptibility testing (DST) and for evaluation of the contribution of re-infection to the epidemiology and pathogenesis of this disease are discussed.

## Methods

### Study population

All consecutive newly registered adult inmates (≥15 years of age) with pulmonary TB admitted to a prison TB hospital near Tbilisi, Georgia from February 2001 to March 2004 were enrolled. All the TB patients included in our study were held in different detention centres and were only referred to the TB prison hospital after they were diagnosed with TB. TB history of study patients was according to the World Health Organization (WHO) guidelines [9]. TB notification rates in the general population during the study period was on average 117.3 cases per 100 000 population (2001–2003) [10]. The HIV infection rate in the Georgian population is <0.2% [11] and 1% among hospitalised TB patients [12]. Demographic data, including sex, age as well as date of diagnosis, clinical diagnosis, and treatment history, were obtained by review of medical and laboratory records. The study was approved by the Ministry of Justice of Georgia.

### Samples and cultures

Three sputum samples were collected under strict supervision at the TB hospital from each of the patients within one week before the initiation of anti-TB treatment, which were collected as part of the routine patient investigation. Each sputum sample from all the patients studied was decontaminated by the modified Petroff's method [13] and cultured on Löwenstein-Jensen (L-J) medium at the National TB Reference Laboratory in Tbilisi, Georgia. The cultures were incubated at 37°C and read weekly for growth for a maximum period of 8 weeks. Identification of the primary isolates was done by classical methods.

### Drug-susceptibility testing

DST was done on all *M. tuberculosis *isolates at the Prince Leopold Institute of Tropical Medicine (ITM) in Antwerp, Belgium by the proportion method on L-J medium containing 0.2 μg/ml isoniazid (INH), 40 μg/ml rifampicin (RIF), 4 μg/ml streptomycin (SM) and 2 μg/ml ethambutol (EMB) [14].

### DNA extraction

DNA was extracted either by boiling bacterial suspensions for 5 min (MIRU-VNTR) or as previously described (RFLP and MIRU-VNTR) [15].

### DNA fingerprinting

DNA fingerprinting of all *M. tuberculosis *isolates was performed by the IS*6110*-RFLP method [15]. Typing of isolates was done blinded i.e. only ITM culture numbers were used to identify isolates during DNA fingerprinting. Patient identities were only revealed when comparing the patterns (BioNumerics, version 3.0; Applied Maths, Sint-Martens-Latem, Belgium). DNA fingerprinting patterns from samples collected over the same period of time were reviewed to detect potential laboratory cross-contaminations or errors.

Secondary typing by MIRU-VNTR was performed on isolates from 30 patients with differences in their IS*6110*- hybridisation patterns and on isolates from another 30 patients with identical IS*6110*-RFLP patterns (controls), using 15 MIRU-VNTR loci selected from a wider set of loci based on their variability in unrelated isolates and stability in clonally related isolates [16-21]. These loci have been found to be more discriminative and better for use in molecular epidemiology studies of TB (Supply *et al*., in preparation) than the 12 previously described loci [20], and they will be therefore proposed for standardisation. The number of repeats in the 15 target loci was determined after multiplex PCR with fluorescently labelled primers against regions flanking the repetitive sequences, electrophoretic separation and sizing of the PCR products using an ABI 3730 XL sequencer [2,19,22]. Specific precautions were taken to avoid and control cross contamination.

### Steps taken to minimise laboratory error

To avoid swapping of sputum samples between patients, containers were pre-labelled with patient identities before sample collection. To minimise laboratory cross-contamination during decontamination and culture of samples, work was done in laminar flow cabinets, and only a limited number of specimens were processed at a time. In addition, study samples were received in batches both in Georgia and Belgium and were processed separately from all other samples received by the laboratory.

To minimise the risk of cross contamination during sample preparation for MIRU-VNTR typing, sample preparation for PCR, and the addition of DNA was done in a laminar flow cabinet. The H37Rv *M. tuberculosis *strain and water were included in each experiment as positive and negative controls, respectively. Reagent contamination could not be detected as all the negative controls were negative on amplification, and the correct number of repeats and no double alleles were detected from the positive controls.

### Statistical methods

Analysis of variance (ANOVA) was used to compare continuous variables and the χ^2 ^test was used for comparisons of proportions between test groups. The analyses were conducted using the Statistical Package for Social Sciences (SPSS version 14.0). A p value < 0.05 was considered significant.

## Results

### Mycobacterial cultures and patient characteristics

**Figure 1:**
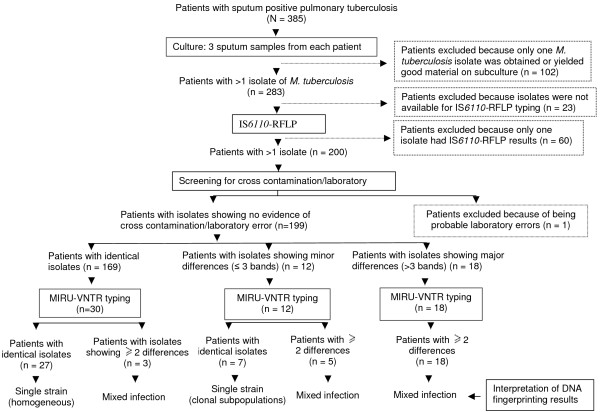
Schematic diagram showing the grouping of patients according to culture results and subsequent IS*6110*-RFLP and MIRU-VNTR typing.

Of the 385 eligible subjects, 186 patients were excluded because: only one sample was culture-positive for *M. tuberculosis *or yielded a good subculture upon receipt in Antwerp (102), only one isolate had IS*6110*-RFLP results (60), isolates were not available for IS*6110*-RFLP fingerprinting after shipment (23), and probable laboratory error (1). The conclusion on the latter case was based on the fact that one of the isolates from the latter patient had identical DNA fingerprinting patterns with an isolate from another patient processed on the same day. Thus, 199 patients with more than one *M. tuberculosis *isolate with IS*6110*-RFLP results were included in the analysis (Figure 1). All the study subjects were male, and the median age of the 198 patients with available age was 30 years (range, 20 to 63 years). Of these, 134 patients were new cases and the remaining 65 patients were retreatment cases. There was no statistically significant difference in the age (p = 0.301) or retreatment types (p = 0.485) of patients that were excluded from the study and those that were included.

### Detection of mixed infections

#### *IS6110*-RFLP screening

All sets of *M. tuberculosis *pre-treatment isolates available from 199 patients were genotyped by IS*6110*-RFLP. The number of IS*6110 *copies among these isolates ranged from 5 to 18 (median, 11 bands). Isolates from 169 patients showed identical patterns within each set, suggesting infection by a single strain. In contrast, isolates from 12 patients (patients 19–30) showed minor differences in IS*6110*-RFLP patterns within their respective sets ranging from 1 to 3 bands indicative of heterogeneous subpopulations, whereas isolates from 18 patients showed major differences of more than 3 bands suggestive of infection by multiple strains (mixed infection). Among the latter, isolates from 7 patients (patients 1–7) showed very distinct IS*6110*-RFLP patterns. Of the remaining 11 patients (patients 8–18), at least one isolate appeared to be a mixture of two different *M. tuberculosis *strains, as evidenced by possession of multiple overlapping bands compared to the other isolate(s) from the same patient (see Additional file 1, Table 2).

**Table 1:** Patient and drug susceptibility results of pre-treatment isolates from patients with variant DNA fingerprinting patterns

**Patient no.**	**Retreatment type**	**Age (years)**	**Isolate no.**	**Drug susceptibility testing**			
				
				**INH**	**RIF**	**SM**	**EMB**
**Patients infected with multiple (mixed) *M. tuberculosis *strains**							
*Patients with incomplete DST results*							
2	New case	23	a	NT	NT	NT	NT
			b	S	S	S	S
*Patients with only pan-susceptible isolates*							
6	New case	22	a + b + c	S	S	S	S
7	New case	36	a + b + c	S	S	S	S
8	New case	38	a + b	S	S	S	S
12	New case	41	a + b + c	S	S	S	S
17	New case	57	a + c	S	S	S	S
18	New case	34	a + c	S	S	S	S
19	New case	24	b + c	S	S	S	S
30	New case	NA	a + b + c	S	S	S	S
*Patients with identical drug resistant isolates*							
5	New case	26	a + b	R	S	S	S
11	New case	29	a + b	S	S	R	S
10	New case	27	a + b + c	S	S	R	S
24	New case	58	b + c	S	S	R	S
33	New case	23	a + b	S	S	R	S
29	Return after default	29	a + b	S	S	R	S
22	Previously received non-official TB treatment	27	a + b	S	S	R	S
16	New case	29	a + b	R	S	R	S
31	Relapse case	38	a + b	R	R	R	R
3	New case	44	a + b+ c	R	R	R	R
32	Failure case	21	b + c	R	R	R	R
*Patients with different drug resistant isolates*							
14	New case	41	b	S	S	S	S
			c	**R**	S	S	S
13	New case	51	a	R	S	S	S
			c	R	S	**R**	S
4	New case	36	a	S	S	**S**	S
			b	S	S	**R**	S
1	Previously received non-official TB treatment	50	a	S	S	R	S
			b	**R**	S	R	S
			c	**R**	S	R	S
15	Return after default	29	a	R	S	S	S
			b	R	S	**R**	S
			c	R	S	**R**	S
9	New case	24	a	R	S	R	S
			c	**R?**	S	R	S

**Patients infected with clonal subpopulations of the same *M. tuberculosis *strain**							
*Patients with only pan-susceptible isolates*							
25	New case	25	b + c	S	S	S	S
20	Relapse case	27	a + b + c	S	S	S	S
*Patients with identical drug resistant isolates*							
21	New case	43	b + c	S	S	R	S
26	New case	37	b + c	S	S	R	S
27	New case	27	a + b	S	S	R	S
28	New case	40	a + b + c	S	S	R	S
23	New case	32	a + b + c	R	S	R	S

Definitions of abbreviations: INH = isoniazid; RIF = rifampicin; SM = streptomycin; EMB = ethambutol, S = Susceptible; R = Resistant; R? = borderline resistance; NT = not tested; a = first pretreatment sputum; b = second pretreatment sputum; c = third pretreatment sputum; NA = not available.

**Table 2:** Results of 33 patients with multiple pre-treatment isolates showing variant DNA fingerprinting patterns

Patient no.	No. of bands different by IS*6110*-RFLP	No. of loci with different/double alleles	Interpretation
1	>3	10	Mixed infection
2	>3	10	Mixed infection
3	>3	12	Mixed infection
4	>3	14	Mixed infection
5	>3	10	Mixed infection
6	>3	11	Mixed infection
7	>3	13	Mixed infection
8	>3	8	Mixed infection
9	>3	11	Mixed infection
10	>3	15	Mixed infection
11	>3	10	Mixed infection
12	>3	12	Mixed infection
13	>3	8	Mixed infection
14	>3	7	Mixed infection
15	>3	9	Mixed infection
16	>3	10	Mixed infection
17	>3	9	Mixed infection
18	>3	12	Mixed infection
19	1	5	Mixed infection
29	1	4	Mixed infection
30	3	7	Mixed infection
31	0	2	Mixed infection
32	0	7	Mixed infection
33	0	10	Mixed infection
22	1	4	Mixed infection
24	1	13	Mixed infection
20	1	0	Identical/subpopulations
21	1	0	Identical/subpopulations
23	1	0	Identical/subpopulations
25	1	0	Identical/subpopulations
26	1	0	Identical/subpopulations
27	1	0	Identical/subpopulations
28	3	0	Identical/subpopulations

Patients are arranged according to the interpretation

#### MIRU-VNTR typing

To study the observed heterogeneity further, all pre-treatment isolates from 30 patients with major (patients 1–18) and minor (patients 19–30) IS*6110*-RFLP differences were typed by MIRU-VNTR using 15 independent loci. In addition, all pre-treatment isolates from 30 patients with identical IS*6110*-RFLP patterns, presumably infected with a single strain, were also typed by MIRU-VNTR as controls. These control isolates were selected to cover the spectrum of IS*6110 *profiles among the different IS*6110*-RFLP clusters and unique isolates found among the 169 sets of isolates with conserved fingerprints within a set.

Consistently, respective isolates from the 18 patients with major IS*6110 *band differences were all found to have very distinct MIRU-VNTR profiles as well, corroborating the conclusion of mixed infection. More detailed analyses of the MIRU-VNTR results of these 18 patients showed that for some of them, different strains could be detected only in different sputum specimens, whereas for other patients the different strains were found to be simultaneously present within one sample. For example, isolates from two of these patients (patients 4 and 5) displayed MIRU-VNTR profiles differing by 10 and 14 loci respectively, and all showed a single allele per locus. This observation provides evidence for the presence of a single strain per sample, but different from the strain from another specimen of the respective patient. Isolates from 14 other patients from this group (patients 1–3, 6–8, 11–18) showed a combination of single alleles differing among the respective pre-treatment samples, and double alleles detected in at least three loci (see Additional file 1, Table 2). One of the two alleles in these loci systematically corresponded to the single allele detected in the same locus of the other isolate(s) from the same patient, or the same double alleles were detected in both isolates. This finding indicates the simultaneous presence of at least two distinct strains, which can be detected in one or more sputa. Furthermore, even triple alleles were reproducibly amplified from three loci of both isolates from patient 10, suggesting the simultaneous presence of three distinct strains. This conclusion was corroborated by the detection of double alleles at three other loci of one of the isolates, none of which was detected in the same locus of the other isolate from that patient. A similar phenomenon was also noticed for both isolates of patient 9, but without detection of any triple allele.

Similarly, the respective isolates from 5 of 12 patients with minor IS*6110 *band differences showed different and/or double alleles in at least three MIRU-VNTR loci, indicating mixed infections. Interestingly, mixed infections as defined on the same MIRU-VNTR basis were also revealed among 3 of the 30 patients whose isolates had identical IS*6110 *fingerprints. Detection of mixed infections by MIRU-VNTR typing among patients with isolates showing identical IS*6110*-RFLP patterns is not surprising because the former method includes an amplification step and a more sensitive, fluorescence-based, detection system.

Identical MIRU-VNTR profiles were obtained among the isolates from the 7 remaining patients with minor IS*6110 *band differences (mostly one band, at most 3 bands), and from the 27 remaining control sets with conserved IS*6110 *fingerprints. In keeping with previous studies [2,23,24], the former group was conservatively assigned to IS*6110 *clonal variants from one original infecting strain and were thus defined as clonal subpopulations, while the latter group corresponded to cases of infection by a single strain, as defined by both typing methods used.

Based on IS*6110*-RFLP data on all isolates and MIRU-VNTR data on isolates from 60 patients we found infection with a single *M. tuberculosis *strain either homogeneous or with some clonal subpopulation in 173 (86.9%) of 199 patients, and mixed infection with two or three distinct strains in 26 (13.1%) patients. Crucially, using the above rules for identification of mixed infections (detection of double alleles in multiple loci), analysis of a single pre-treatment isolate per patient would have led to missed mixed infection in 14 of these cases using MIRU-VNTR typing. Analysis of a single pre-treatment isolate per patient would have even led to missed mixed infection in all cases using standard IS*6110*-RFLP, as no evidence of strain mixture could be detected in single patterns.

Twenty-one of the 26 patients with mixed infection were new TB cases, 1 was a treatment failure case, 3 returned after default while 1 had previously received unofficial TB treatment (Table 1). The distribution of the treatment history of TB among these cases was not significantly different from the distribution among those infected with a single homogeneous or heterogeneous strain (χ^2^, p = 0.117).

#### Drug susceptibility testing

Phenotypic DST classified 80 of 199 patients as having been infected with pan-susceptible isolates and 20 patients with multi-drug resistant (MDR) isolates. The remaining 99 patients were infected with isolates that were resistant to at least one drug, but not MDR.

From 25 out of 26 mixed infection cases with known DST results for all isolates, 8 patients were infected with only pan-susceptible strains, while the other cases showed resistance in at least one of the isolate. Out of these, clear differences in resistance patterns among isolates from the same patient were observed only in 5 cases (patients 1, 4, 13, 14, 15, Table 1). In another case (patient 9) however, one isolate showed a clear resistance to INH whereas a borderline result was obtained in the other. Isolates from patients 1 and 14 differed in their susceptibility to INH, which was confirmed by sequencing of the *kat*G and *inh*A genes (data not shown). The resistant isolate for patient 9 was confirmed by sequencing i.e. presence of a novel T→C (in contrast to the previously reported T→A or G) mutation at position -8 of the *inh*A gene [24]. Interestingly, a mixture of both a wild type (T) and the novel mutation (C) was obtained at the same position for the isolate with a borderline result, thereby corroborating our MIRU-VNTR findings. Patient 9 and 14 were new cases, while patient 1 had previously received unofficial anti-TB treatment. Difference in SM resistance was observed in the remaining 3 patients (4, 13, 15), but this was not investigated further.

In contrast to the above mixed infection cases with isolates showing different DST patterns, it is remarkable to note that the remaining 11 patients were infected with two or three different *M. tuberculosis *strains that showed exactly the same DST pattern: 1 mono-INH-resistant, 6 mono-SM-resistant, 2 INH + SM-resistant and 3 MDR. Eight of these patients were classified as new cases (Table 1).

Finally, among the 7 patients with clonal subpopulations, none showed differences in DST among their respective sets of isolates whereas among the patients with genetically homogeneous bacterial populations, one retreatment and three new cases showed a difference in SM among the isolates (data not shown).

## Discussion

This report simultaneously assessed the validity of two interdependent postulates on which standard analytical schemes rely: (i) that a TB patient can only be infected with a single homogeneous *M. tuberculosis *strain at any given time, and (ii) that an isolate from a single sputum specimen is representative of the total bacillary population in a patient. Therefore, we systematically compared the genetic relatedness of *M. tuberculosis *isolates from multiple sputum samples collected prior to the initiation of anti-TB therapy from each of 199 smear-positive inmates admitted to a prison TB hospital. By using two independent genotyping methods to differentiate strains, we detected infection with two or even three distinct *M. tuberculosis *strains in 13.1% of the samples analysed. There was no significant difference in the proportion of retreatment cases between the excluded and included patients.

The mixed infection rate observed in this prison population can not be extrapolated to the general population because of overcrowding and higher incidence of TB in the prisons compared to the general population (5,995/100 000 vs. 155/100 000 population, respectively) [26]. However, we believe that the so called "cheating" (*i.e*. prisoners attempt to submit sputa mixed with that of other prisoners suspected of having smear-positive TB, so that they can be diagnosed with TB and transferred to the prison TB hospital with better living conditions than in other detention centres) had a low influence on our estimation of this phenomenon, if any, due to the strict and active surveillance by an aware staff at the sputum collection step inside the TB hospital. Likewise, laboratory cross-contamination is an unlikely explanation for the high frequency of mixed infection detected because of the specific precautions taken.

The use of IS*6110*-RFLP-typing as an initial screening method may have led to some underestimation of mixed infection because this method has inherent limitations to detect mixed infections within a single isolate since various bands in a given profile can represent one or more strains. Moreover, it remains unclear to what extent low ratios of one of the strains present in a mixture are reflected in low-intensity bands [27] or not detected at all. The latter is evidenced by the detection of mixed infections by MIRU-VNTR among 3 (10%) of 30 patients with isolates that showed identical IS*6110*-RFLP patterns but double alleles in multiple loci within one isolate by MIRU-VNTR. By extrapolation, this suggests that up to 14 (7.0%) mixed infections might have been additionally detected among the other 139 patients with isolates that had identical IS*6110*-RFLP patterns if they were also tested by MIRU-VNTR. There was no statistically significant difference in retreatment type between new and previously treated cases among the patients whose isolates were only typed by IS*6110*-RFLP and those whose isolates were additionally typed by MIRU-VNTR (χ^2^, p = 0.15). Finally, some mixed infections could have remained undetected by MIRU-VNTR typing itself although this PCR-based method is able to detect ratios of a given strain as low as 1:99 [28].

Detection of genetically distinct strains among multiple pre-treatment sputum samples, as well as within a single sputum specimen might reflect separate lesions in the lungs containing different *M. tuberculosis *strains and opening simultaneously or consecutively as also suggested from a previous study [29]. Regardless of the explanation, it is crucial to note that if only the first pre-treatment sample was analysed by standard IS*6110 *fingerprinting or by MIRU-VNTR typing, none or only about half of the mixed infection cases detected by analysis of multiple pre-treatment samples (14 cases vs. 26 cases) would have been identified. These observations imply that analysis of a single isolate, especially in high incidence settings may underestimate the actual heterogeneity of the bacillary population in the host.

It is relevant to observe that the 13.1% of mixed infections detected in this prison population is relatively close to the frequency of 19% recently reported in the study of Warren *et al*. in a general population of a setting with an incidence 1000/100 000 population [3]. Although the two values are not directly comparable as this latter evaluation was limited to the detection of patients simultaneously infected with strains of both the Beijing and non-Beijing lineages, we predict that their value is likely an underestimation as only single isolates per patient were analysed in that case. Similarly, previous studies in high incidence settings might have overestimated the contribution of reinfection vs. relapse due to undetected initial mixed infection [7,29]. Our observations imply that for specific research studies analyses of several isolates from different sputum samples at each disease episode (before and after treatment), especially in high TB incidence settings might be helpful in distinguishing true reinfection vs. relapse and/or mixed infection, preferably using a PCR-based typing method like MIRU-VNTR.

From the 26 proven mixed infection cases in our study, 30% harboured only pan-susceptible strains, whereas 70% showed any resistance in at least one of the isolates. In only 6 cases was mixed infection reflected in a variant DST profile. Remarkably, the remaining patients were infected with two or three strains seemingly showing identical resistance profiles. Although most of these patients were new cases according to WHO definitions [9], we can not completely exclude the possibility that they might have taken TB drugs for less than one month, and therefore both strains might have acquired resistance as a result of the same drug pressure. On the other hand, independent infection with two or three strains showing exactly the same resistance profile for each of so many patients is very doubtful as well, even in a setting with a high rate of drug-resistant TB. Most probably, such frequent observations of identical resistance profiles among respective isolates from these mixed infections reflect the systematic presence of both a susceptible and a resistant strain in the corresponding specimens. This systematic duality was evidenced by the systematic detection of double alleles in the MIRU-VNTR patterns in the isolates from all these cases but one. In such conditions, the simultaneous growth of a (more) resistant strain will mask that of susceptible (or partly resistant) strains in DST assays either completely or partly resulting in either resistant isolates or isolates with borderline results (patient 9). In such situations, culturing and DST of single pre-treatment sputum had generally no predictable adverse consequences for the appropriateness of the treatment regimen of the respective patients.

As mentioned above, variant DST profiles were detected as a result of mixed infections in only 6 (3.0%) of the 199 patients. This finding lends support to previous reports that initial mixed infections may actually be responsible for changes in DST patterns in isolates of some patients [29-31].

In general, our findings suggest that single-isolate analyses can be used for routine DST in most settings, except for some high drug resistant-TB-prevalent settings. However, for specific research studies like treatment evaluation and clinical trials, testing multiple isolates from different sputum samples at each disease episode could help in determining the respective contribution of mixed infection and reinfection versus relapse with gradual development of drug resistance, especially by PCR-based typing methods such as MIRU-VNTR.

Although the high rates of mixed infection in this prison setting can not be extrapolated to the general population with a lower risk of TB transmission, these findings nonetheless indicate that an initial infection is unable to provide protection against a subsequent infection in these populations, which have implications for the development and trials of new vaccines [3]. Because higher rates of mixed infection imply possible higher rates of super infection, the protective effect of an initial infection against a subsequent infection may be even lower than expected. This parameter needs to be taken into account in the development of new prophylactic approaches.

## Conclusion

This study has demonstrated that different pre-treatment sputum samples from a patient can harbour distinct *M. tuberculosis *strains. In addition, the study has shown the occurrence of varying DST patterns among multiple pre-treatment isolates, which might indicate mixed infection or ongoing acquisition of drug resistance. Our findings are important for the correct interpretation of molecular epidemiology data in follow-up studies in high incidence settings and in treatment evaluations.

## Competing interests

The author(s) declare that they have no competing interests.

## Authors' contributions

ICS: Performed DNA fingerprinting, evaluated the data, drafted and reviewed the manuscript. LJ: Co-ordinated the study and helped in drafting the manuscript. NS: Supervised sample collection, culture and identification of mycobacteria. EW: Participated in MIRU-VNTR typing. FP: Conceived of the study, participated in its design and reviewed the manuscript. PS: Supervised MIRU-VNTR typing, evaluated the data, helped in drafting and revising the manuscript. LR: Conceived of the study, participated in its design, co-ordination and evaluation of data, and helped in drafting the manuscript. All authors read and approved the final manuscript.

## Supplementary Material

Additional File 1 DNA fingerprinting results of pre-treatment *M. tuberculosis *isolates with variant patterns from each of the respective 33 patients. a = first pre-treatment sample; b = second pre-treatment sample; c = third pre-treatment isolate from each patient; d = MIRU-VNTR loci are listed according to their position (in kilobases) on the H37RV genome. Alternative designations are indicated in parentheses. For isolates with minor IS*6110*-RFLP variations, arrows indicate additional band (s); ND = not determined; *A third allele was detected at the respective locus. Al1, Al2 = Allele 1 and Allele 2, respectively. 2S or 3S = variant alleles in locus MIRU 04, similar to those in the H37RV and BCG genomes [20]. Click here for file
