# Supplementary figures and images for: Mixed infection and clonal representativeness of a single sputum sample in tuberculosis patients from a penitentiary hospital in Georgia
Source: Respir Res. 2006 Jul 17;7(1):99. doi: 10.1186/1465-9921-7-99 (PMC1538999; doi:10.1186/1465-9921-7-99)

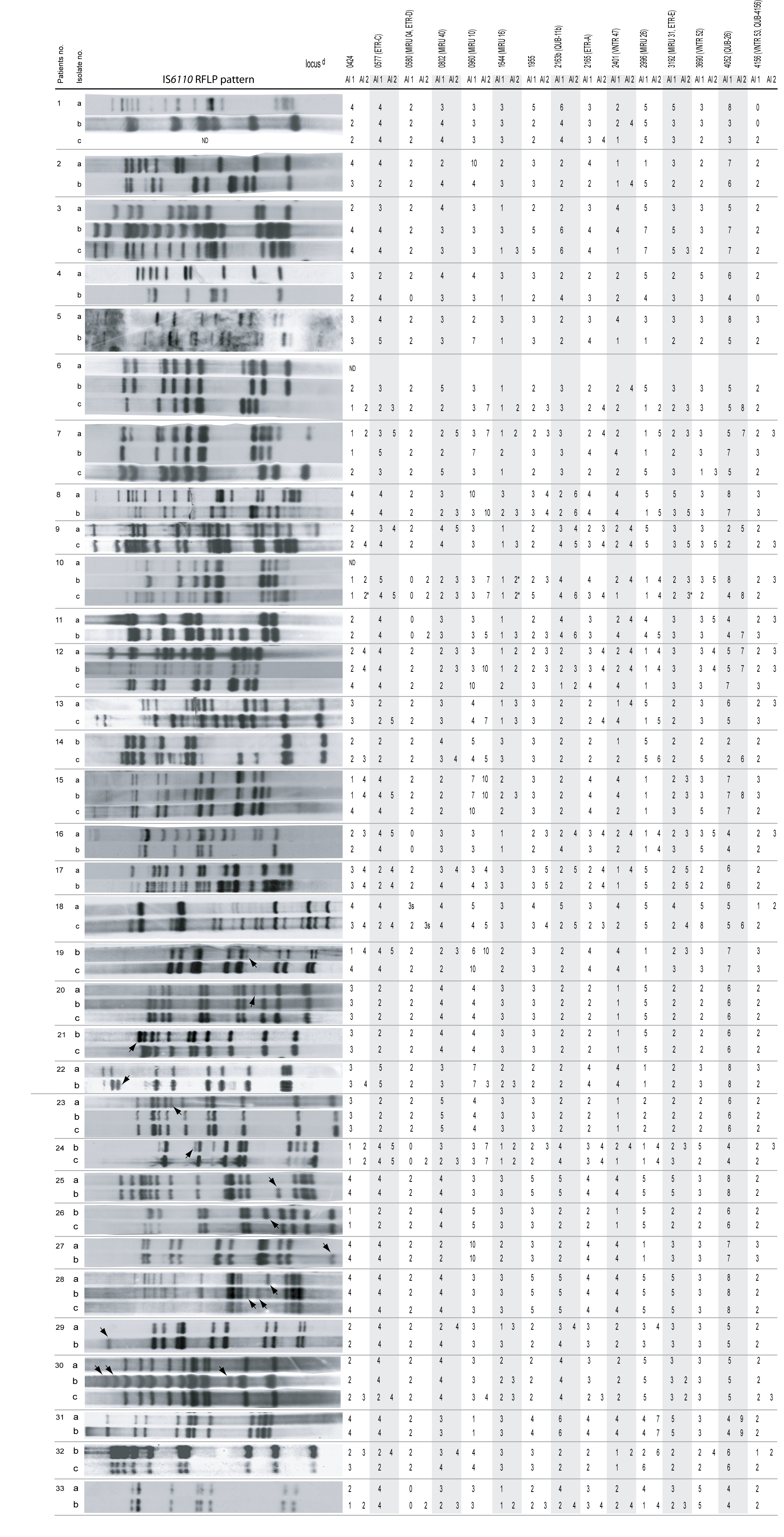

Supplement: Additional File 1 — DNA fingerprinting results of pre-treatment M. tuberculosis isolates with variant patterns from each of the respective 33 patients. a = first pre-treatment sample; b = second pre-treatment sample; c = third pre-treatment isolate from each patient; d = MIRU-VNTR loci are listed according to their position (in kilobases) on the H37RV genome. Alternative designations are indicated in parentheses. For isolates with minor IS6110-RFLP variations, arrows indicate additional band (s); ND = not determined; *A third allele was detected at the respective locus. Al1, Al2 = Allele 1 and Allele 2, respectively. 2S or 3S = variant alleles in locus MIRU 04, similar to those in the H37RV and BCG genomes [20]. [file 1465-9921-7-99-S1.jpeg]
